# Wild Steps in a semi-wild setting? Habitat selection and behavior of European bison reintroduced to an enclosure in an anthropogenic landscape

**DOI:** 10.1371/journal.pone.0198308

**Published:** 2019-11-07

**Authors:** Pil Birkefeldt Møller Pedersen, Joanna B. Olsen, Brody Sandel, Jens-Christian Svenning

**Affiliations:** 1 Department of Bioscience, Section for Ecoinformatics & Biodiversity, Aarhus University, Aarhus, Denmark; 2 Department of Bioscience, Center for Biodiversity Dynamics in a Changing World (BIOCHANGE), Aarhus University, Aarhus, Denmark; University of Tasmania, AUSTRALIA

## Abstract

Recently, several wild or semi-wild herds of European bison have been reintroduced across Europe. It is essential for future successful bison reintroductions to know how the European bison use different habitats, which environmental parameters drive their habitat selection, and whether their habitat use and behavioural patterns in new reintroduction sites differ from habitats where European bison have been roaming freely for a long time. Here, we address these questions for a 40-ha enclosed site that has been inhabited by semi-free ranging European bison since 2012. The site, Vorup Meadows, is adjacent to the Gudenå river in Denmark and consists of human-modified riparian meadows. During 2013 we monitored the behavioural pattern and spatial use of the 11 bison present and in parallel carried out floristic analyses to assess habitat structure and food quality in the enclosure. We tested habitat use and selection against environmental parameters such as habitat characteristics, plant community traits, topography, and management area (release area vs. meadow area) using linear regression and spatial models. The bison herd had comparable diurnal activity patterns as observed in previous studies on free-roaming bison herds. Topography emerged as the main predictor of the frequency of occurrence in our spatial models, with high-lying drier areas being used more. Bison did not prefer open areas over areas with tree cover when accounting for habitat availability. However, they spent significantly more time in the release area, a former agricultural field with supplementary fodder, than expected from availability compared to the rest of the enclosure, a meadow with tree patches. We wish to increase awareness of possible long-term ethological effects of the release site and the management protocols accomplished here that might reduce the ecological impact by the bison in the target habitat, and thereby compromise or even oppose the conservation goals of the conservation efforts.

## 1. Introduction

Megaherbivores and other large herbivores are increasingly acknowledged for their importance in shaping ecosystems in terms of physical structure [1, 2], trophic structure [1, 3], vegetation composition and diversity [1, 4–6] and biochemistry [1, 7] through feeding behaviour and pressure, trampling activity, seed dispersal, droppings and eventually as carcasses. Accordingly, loss and absence of large herbivores can have significant and far-reaching effects [8–11].

In the Late Pliocene and Early Pleistocene the genus *Bison* appeared widely in the temperate regions of Asia and Europe giving origin to different forms of bison including *Bison schoetensacki*. *Bison schoetensacki* was present in Western Europe until the Upper Pleistocene and is thought to be a sister species to the modern European bison, *Bison bonasus* [12], which emerged in southern Caucasus after the Last Glacial Maximum (22kya) [13] and inhabited northern Germany and southern Scandinavia approximately 10kya [14]. By mid-Holocene (5kya) *Bison bonasus* had disappeared from Western Europe, and later it also disappeared from Central and Eastern Europe [15]. The decline of *Bison bonasus* is thought to be caused by hunting [13] and increasing intensification of agriculture leading to habitat destruction and fragmentation forcing the escaping bison into the forest of Eastern Europe [13]. By the late 1800s, there were only two European bison populations left, and the last wild bison died of disease in 1927 [15]. After World War I, a breeding program was initiated in 1920ies based on 12 individuals out of the 54 individuals surviving in zoos across Europe [13].

In the last few decades, European bison (*Bison bonasus*) have been reintroduced to various habitats across Europe, ranging from coastal dunes in the Netherlands (e.g. [16, 17]) over mountainous mosaic landscapes in the French Alps [18] and in Germany [19] to lowland peri-urban meadows [20]. Reintroduction areas not only represent different habitats, they also represent different degrees of human modification, with some reintroduction sites being naturally disturbed coastal dunes (e.g. [17]), private commercial plantation (e.g. [19]) or areas still ditched and drained due to upland agricultural use [20]. The conservation goal of these reintroductions is often twofold, with one focus being the protection of the largest extant wild, native herbivore in Europe, European bison [21], and the other focus being ecological restoration focusing on restoring trophic top-down interactions and associated cascades as well as non-feeding related processes of the European bison (i.e. trophic rewilding [22]) [17].

Reintroductions have until recently primarily occurred in forested areas [19] as European bison have been thought to be a forest specialist originating from closed forest habitats. However, recently more studies report that bison originally roamed in mosaic landscapes foraging on grassland plant species [14, 23–26]. The extensive reintroduction work has so far paid off, as there now are 1647 bison in captivity, 400 semi-free bison and 4009 free-living, mainly located in Poland, Belarus, Russia, and Caucasus [27] and the IUCN red list category has been moved from ‘endangered’ to ‘vulnerable’ in 2008 [28]. However, challenges still remain as the free-living bison are distributed on 40 small and rather isolated populations with no or little genetic exchange [27]. None of these wild populations are considered to be self-sustaining [29].

Despite the many reintroductions of European bison across Europe, there is still no clear roadmap for how to successfully reintroduce bison. This is even more challenging in recent reintroductions in anthropogenic landscapes in Western Europe where habitats often are size-restricted and multifunctional [18, 19, 30]. Consequently, it is even more important to match the habitat requirements of bison to the reintroduction site in order for it to be successful. Reintroduction efforts should, therefore, undergo evaluation to help fill in the many knowledge gaps e.g. do the bison thrive in the specific enclosure and habitat, how do they use it and what management protocols are optimal for trophic rewilding?

In Denmark two small semi-wild herds have been established; in the riparian meadows of Gudenå river close to the city Randers in Jutland since 2010 [20] and since 2012 in the forests of Almindingen on Bornholm Island [31]. The habitat use of two individuals of the herd consisting of 11 individuals on Bornholm has been evaluated using GPS collars [32]. This study will be the first to study the other case in the peri-urban meadow in Jutland.

In this study, we provide an assessment of the daily and seasonal behaviour of the bison and compare it to that of free-ranging herds from Bialowieza Forest [33]. We also investigate the bison herd´s habitat use by linking the occurrence of the bison herd to environmental parameters such as habitat characteristics, plant community traits, topography, and management area (release area with supplementary feeding vs. semi-natural meadow area) to better inform future reintroductions of European bison and rewilding-inspired efforts in terms of assessing suitable habitat and management interventions.

## 2. Material and methods

### 2.1 Study area

**Fig 1:**
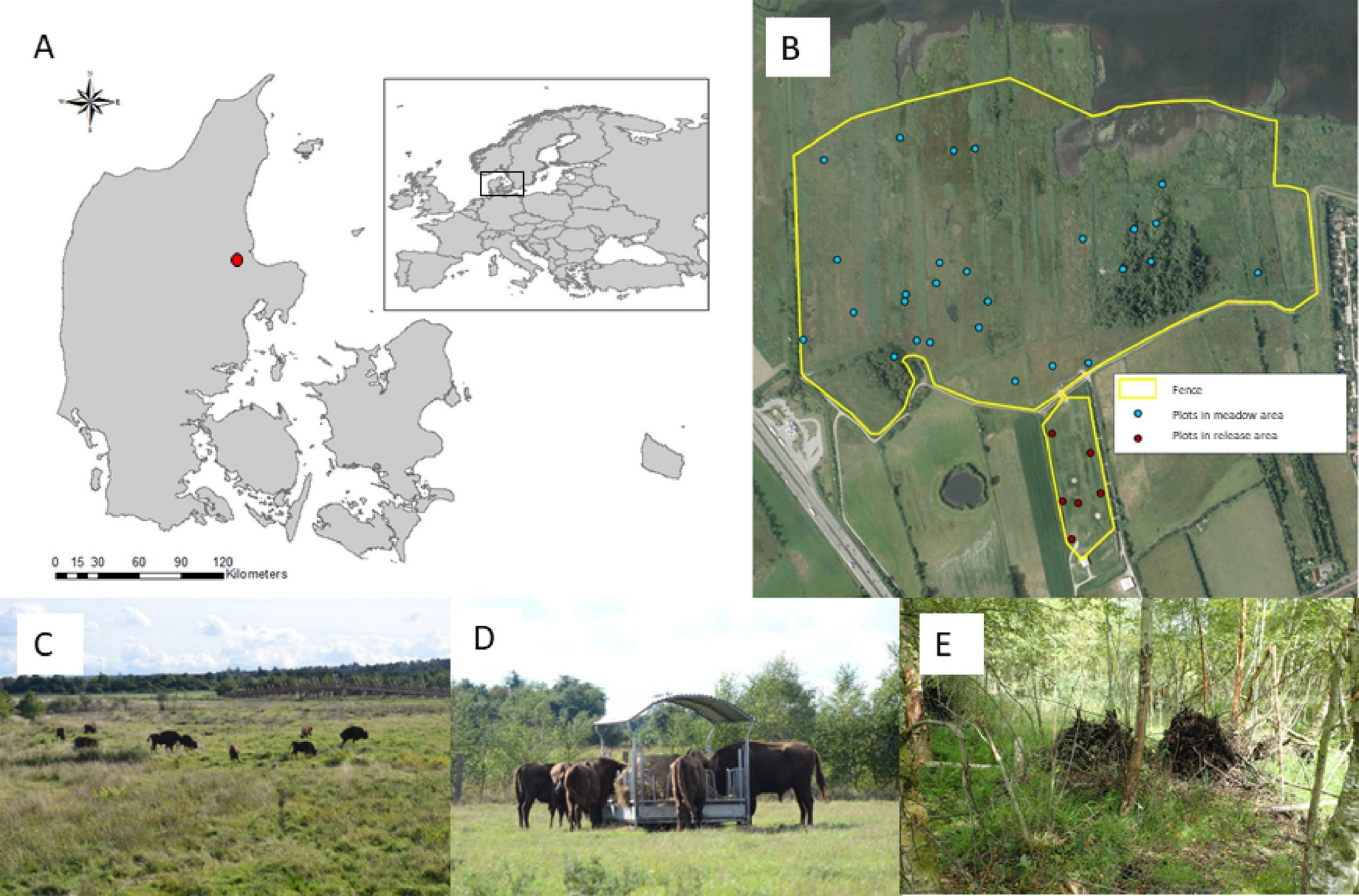
Overview of and photos of the study area. (A)The big map shows Denmark with the study location marked with a red dot and the inserted map shows the location of Denmark in Europe. (B) Ortho photo of the bison enclosure (yellow line) showing the GPS points used for the vegetation analysis within the meadow (blue points) and within the release area (red points). (C) Photo of the bison herd feeding in the meadow. (D) Bison herd feeding at the hay rack in the release area. (E) Photo of the forest habitat in the eastern forest patch.

The study was conducted in a 40 ha large bison enclosure located along the river Gudenå, in the Eastern part of Jutland, western Denmark (Fig 1A). The area mostly consists of wet meadows (79.7%) partly susceptible to flooding, small deciduous forest patches (10.3%), and previously cultivated grassland (5.2%). The two largest tree covered patches (eastern: 2.5ha and western: 0.7ha) (Fig 1B) can provide shelter for the herd during rainy and windy weather conditions and shadow during hot periods. The bison enclosure consists of two areas (Fig 1B), a release area (Fig 1D) and a meadow area (Fig 1C) with tree covered patches (Fig 1E), between which the bison have free access most of the year. Formerly, the release area had been used for hay-harvest followed by cattle grazing. The release area contains a permanent stable, hay rack, and a water container (Fig 1D). The bison enclosure is fenced by a 2.3m external fence netting and an internal electric single-wire fence (Gallagher Mx75500, 7.4 kV, 0.85cm above ground). The enclosure is located 0–3 m above sea level and is still being drained by use of drainpipes and ditches, though less effectively as during former land use practices. Roe deer (*Capreolus capreolus*) have been spotted inside the bison enclosure, as well as hares (*Lepus europaeus*), stoat (*Mustela erminea*) and foxes (*Vulpes vulpes*).

### 2.2 Study animals

Here we study 11 European bison of the lowland line (*Bison b*. *bonasus*) (1 bulls, 4 cows, and 6 female juveniles) that arrived at the bison enclosure in the summer of 2012. When the bison first arrived, they were initially kept in the release area, which is positioned furthest away from the open water and highest above sea level. During winter (October-March) supplementary fodder (silage and compound feed) is provided in the release area to fulfil Danish legal animal ethics, e.g. animals should stay in good body condition year-round. Supplementary fodder is also occasionally available during other times of the year as a means to apply veterinary treatment to the bison. According to the recently published framework Trophic Rewilding Advancement in Anthropogenically Impacted landscapes (TRAAIL) [34] enabling categorisation of rewilding efforts based on the degree of self-regulation, this conservation project can be referred to as a partial rewilding project according to the managers ambition of letting the bison have year-round access to the study area, letting them exert their natural functions in order to advance the ecosystem into a self-regulating biodiverse ecosystem, while not supporting natural population dynamics due to the size-restricted area. We note that the project was discontinued in 2019 due to severe parasite infections by common liver fluke, *Fasciola hepatica*, leading to nine out of fourteen animals to be put down. The remainder have been translocated to another rewilding area in Jutland.

### 2.3 Data collection of bison behaviour, habitat use and plant species

The methodology applied to record behaviour and habitat use generally follows the methodology used by Caboń-Rackzyńska et al. 1987 [33] as we wish to compare the results from this study with ours. Observations on behaviour and position were made from April 29 to September 27, 2013. During this period, observations were made 2–3 days a week on average, with a total of 44 observation periods. Each observation period lasted 7–9 hours with a scan-sampling interval of 15 minutes recording behaviour and position. Observation periods were either ranging from dawn until noon, or from noon until dusk, where the herd would lay down for the night. In total, 22 cycles from dawn to dusk were obtained, summing up to 326 observation hours in total and resulting in 1380 observation data points. Temperature and weather conditions were also logged, as well as notes about unusual or relevant events. The whole observational period (April to September) was divided into three seasons (spring, summer, and autumn) based on the average daily temperature (S1 Fig), with the summer period being significantly warmer than the spring and the autumn period (Dunn´s test: p = 0.0001 and p = 0.0004, respectively). Observations were made from outside of the enclosure (sometimes from on-site bird observation towers) and binoculars (Vortex Talon HD 10X42) were used if necessary in order to categorize the behaviour of the bison (observation distance: min.: two meters, max.: 500m). The behaviour observed was divided into four categories (feeding, resting, moving, and other), inspired by previous bison studies [33, 35] (Table 1). Type of behaviour was designated to the entire herd based on the type of behaviour the majority of the herd showed at the logging time. This observational study was approved by the organization responsible for the animals, Randers Rainforest.

**Table 1:** Description of the four different types of behaviour monitored while observing the bison herd.

Behaviour type	Description
Feeding	The animal is standing still or taking slow steps without lifting its head.
Resting	The animal is lying down on the ground ruminating or sleeping.
Moving	The animal is walking or running with its head lifted off the ground and not eating.
Other	The animal is standing still with its head lifted. Nuzzling or scratching, playing or fighting, urinating or defecating autumns under this category.

To map the vegetation in the bison enclosure, 36 vegetation plots were randomly positioned based on random geographic coordinates assigned in R [36] (Fig 1B). Every plot location was found using a Trimble Juno SB GPS, however, three plots were positioned in open water and therefore discarded. Each plot consisted of a circle of 10 meters in diameter according to the guidelines of the Danish National Monitoring and Assessment programme for the Aquatic and Terrestrial Environment (NOVANA) [37]. Plant species were identified on a presence/absence level in each plot. Access to the area and execution of floristic analysis was done in accordance with and approved by the organizations involved, Randers Rainforest and Aage V. Jensens Nature Foundation.

### 2.4 Analysis of behavioural patterns and habitat use

Observational data on behaviour and position were processed in ArcGIS 10.1 [38]. The study area was divided into 20m grid cells (40m^2^). This grid size was chosen based on the observed space used by the 11 bison. The position and type of behaviour were summed for each grid cell across the whole study period to link the spatial habitat use to predictor variables. Type of behaviour was summed for each grid cell across each defined season to analyse behaviour across season. Time spent on feeding, resting and moving was tested against previous findings by Caboń-Rackzyńska et al. [33] on free-living European bison in Bialowieza Forest. This study reported that bison on a daily basis forage 60.4%, rest 31.9%, and move 7.7% during periods with no snow cover. The percentage of each habitat type in each grid was calculated. Spatial analysis was conducted in R [36]. One grid cell was removed from analysis involving frequency of occurrence as we considered it an outlier as bison often were observed here (99 times) due to this grid cell being the physical link between the two management areas (release area vs. meadow area with tree patches) of the enclosure.

We tested the difference in frequency of occurrence among habitat types (cultivated field (release area), meadow, water, and tree covered areas) with a Pearson´s Chi-squared test and difference in frequency of occurrence among habitats when accounting for habitat availability was also tested pairwise with a Pearson´s Chi-squared test. We calculated Jacob´s index, D, [39] which is a food selection index independent of habitat availability as follows: D = (r—p)/(r + p– 2rp), where r is the proportion of habitat used and p the proportion of habitat available. D varies from -1 (strong avoidance) to +1 (strong preference), and values close to zero indicate that the habitat is used in proportion to its availability. Mann Whitney t-tests were used to test if the behavioural pattern or frequency of occurrence differed across management area (release area vs. meadow area). Whether the behavioural pattern of the bison herd differed across season (spring, autumn, and summer) was tested with a Kruskal Wallis test, and significant test results were followed by pairwise test using Dunn test using no p-value correction. Correlation between time spent on a certain behaviour (feeding or resting) and the daily average temperature was tested with a corrected Pearson´s correlation test accounting for temporal autocorrelation. All statistical tests were performed in R [36] and datasets are available in Supporting Information.

### 2.5 Analysis of habitat selection

**Fig 2:**
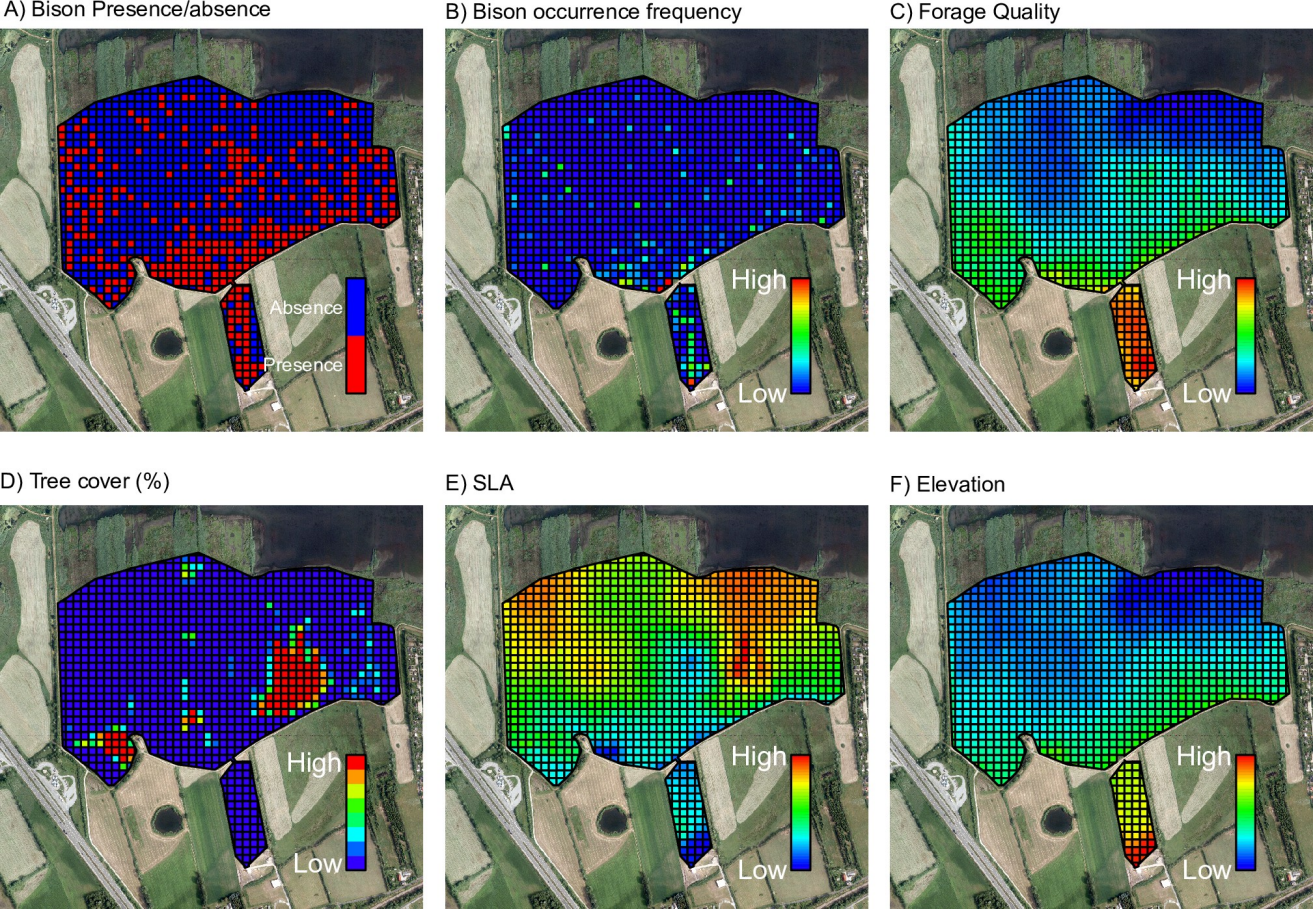
Response and predictor variables used for the spatial linear and logistic regression. Response variables: (A) Response variable: Presence/absence and (B) frequency of occurrence of the bison herd summed across the whole observation period. Predictor variables: (C) forage quality, (D) tree cover, (E) SLA, and (F) elevation.

We considered two response variables (Fig 2A and 2B); the frequency of occurrence of bison in each grid cell in the bison enclosure and presence/ absence of bison in each grid cell in the bison enclosure. Frequency of occurrence was used as a measure for how often the bison herd occur in a certain grid cell. Presence/absence was used as a measure for whether or not the bison herd occur in a certain grid cell. We considered five predictor variables (Fig 2C–2F and Table 2). Three variables describe the local environment: elevation derived from the Digital Terrain Model [40], tree cover, and management area (release area vs. meadow area with tree patches) and two describe variation in the plant community; forage quality for cattle [41] retrieved from BiolFlor [42] and Specific Leaf Area (SLA: leaf area per leaf dry mass) retrieved from LEDA traitbase [43] in order to explain the habitat use by the bison. Elevation was included to gain insight about the role of hydrology on habitat selection as the enclosure ranged from a submerged area close to the river to a dry field furthest away from the river. Tree cover was a relevant variable to include considering the ongoing debate about whether bison prefer open habitats, mosaic-landscapes or closed forests [23]. Management area (release area with supplementary feeding vs. semi-natural meadow area with tree patches) was considered to assess the influence of the enclosure configuration and management protocols as this might affect their habitat selection and behaviour. Forage quality and SLA were included as it is likely that bison prefer areas where plants species provide high forage quality or digestibility, which has been found to increase with increased SLA [44] and might constitute a relatively easy measure predicting where bison impact should be expected.

**Table 2:** Description of the predictor variables used in the spatial linear and logistic regression.

Variables	Description
*Elevation*	The Danish Digital Terrain Model [50] is a measure of the ground elevation and indicated the height of ground and thereby areas of open water or areas most likely to be flooded. The Digital Terrain Model was extracted from Light Detection and Ranging (LiDAR) data.
*Digital Object Model (used only for co-kriging)*	The Danish Digital Object Model is calculated as the difference between Digital Terrain Model and Digital Surface Model and show the height of everything above ground level (trees, shrub, and buildings).
*Tree cover*	The percentage of the habitat consisting of deciduous tree stands, hedges and shrubs. Constructed from ground-truthing and ortho-photos from 2012.
*Management area*	The bison enclosure consists of the release area, a formerly cultivated field, and the meadow area.
*SLA*	SLA was calculated as the product of the Specific Leaf Area (SLA) for each plant monitored on a presence/absence level in a 10m circle in the vegetation plots and interpolated to the entire enclosure using co-kriging with Digital Object Model and elevation.
*Forage quality*	Forage quality was calculated as the product of the forage quality for each plant monitored on a presence/absence level in the 10m circle in the vegetation plots and interpolated to the entire enclosure area using co-kriging with Digital Object Model and Digital Terrain Model. Forage quality for cattle was retrieved from the BiolFlor database [42].

SLA and forage quality were interpolated to the whole study area based on presence/absence data on plant species obtained from the 33 vegetation plots in order to include these variables in the spatial models (Fig 2). We used kriging with the following co-variables; elevation and Digital Object Model (a measure for vegetation structure), which was derived from elevation and Digital Surface Model [40]. Performance of co-kriging was tested using a training subset and a validation subset of vegetation plots and resulted in a prediction of 67.2% for SLA and of 85.1% for forage quality. Co-kriging was performed using the function krige in the R package gstat [45, 46] and based on exponential variogram shape.

The response variable frequency of occurrence was log transformed to ensure normality of the residuals, and all grid cells with zero values (no observations of bison) were removed to avoid skewness and bias in the residuals. One grid cell was removed from analysis involving Frequency of occurrence as we considered it an outlier as bison often were observed here (99 times) due to this grid cell being the physical link between the two management areas (release area vs. meadow area with tree patches) of the enclosure.

The relationship between the occurrence frequency (the number of times the bison herd occurred in the same grid cell) and elevation, tree cover, management area, forage quality and SLA was tested with simple linear regressions for continuous explanatory variables and with Pearson product moment correlation for categorical explanatory variables.

Multicollinearity among predictor variables was tested using Spearman’s rank correlations for variables with a non-linear relationship and Pearson´s correlation between variables with a linear relationship. Multicollinearity was considered a problem among predictor variables when correlations rose above 60%, and therefore management area and forage quality were excluded from the habitat selection models as they were highly correlated with elevation (See S1 Table). Elevation was not discarded as this predictor variable was of highest resolution.

Spatial autocorrelation was tested by evaluating Moran´s I for the lower distance classes of the residuals and considered to be negligible (see S2 Fig). Linear regression models were conducted to test if the predictor variables (elevation, tree cover, and SLA) influenced how often the bison herd occurred in a given grid cell (frequency). Multiple logistic regression models were performed to test if the predictor variables (elevation, tree cover, and SLA) had an effect on whether the bison occurred in the grid cell or not (presence/ absence). All possible combinations of models (eight models for both linear and for logistic models) were fit and ranked according to their relative weight of evidence (using Akaike weights, w_i_) calculated from Akaike’s Information Criterion corrected for small sample size (AIC_c_). Models were assessed according to the strongest support (w_i_) and explanatory power [47]. To evaluate the explanatory power of each individual variable, we calculated model-averaged coefficient estimates (parameter estimates weighted according to support across all eight models) and relative variable importance (RVI, the sum of Akaike weights for each model in which the explanatory variable occur) [47]. Spatial model selection and model averaging were conducted using R package MuMIn [48].

We also looked into an old study assessing the natural food preferences on European bison in Poland [49] to investigate if the results of this study supported a relationship between feeding preference and SLA. We therefore assigned SLA values to all the plant species for which Borowski and Kossak [49] had measured abundance coefficients (D, after German “Deckungswert”) and contacts between bison and plant (n) (bites) and tested the relationship between food preference and SLA (n/D~SLA) with a linear regression.

### 2.6 Ethics statement

The animals were kept in a 40 ha enclosure privately owned by the NGO Aage V. Jensen Nature Foundation. The zoo Randers Rainforest purchased six animals from UNK at Bialowieza National Park and five animals were donated to the zoo Randers Rainforest from another Danish zoo Ree Park–Ebeltoft Safari. The zoo Randers Rainforest was responsible for animal handling, control, release and zookeepers supervised the animals daily. Randers Rainforest has obtained the necessary import permission for releasing the animals. The physical body condition of the animals was regularly checked by the supervising veterinarian of the zoo Randers Rainforest. This study did not imply animal sampling of protected animals.

## 3. Results

### 3.1 Behavioural patterns

**Fig 3:**
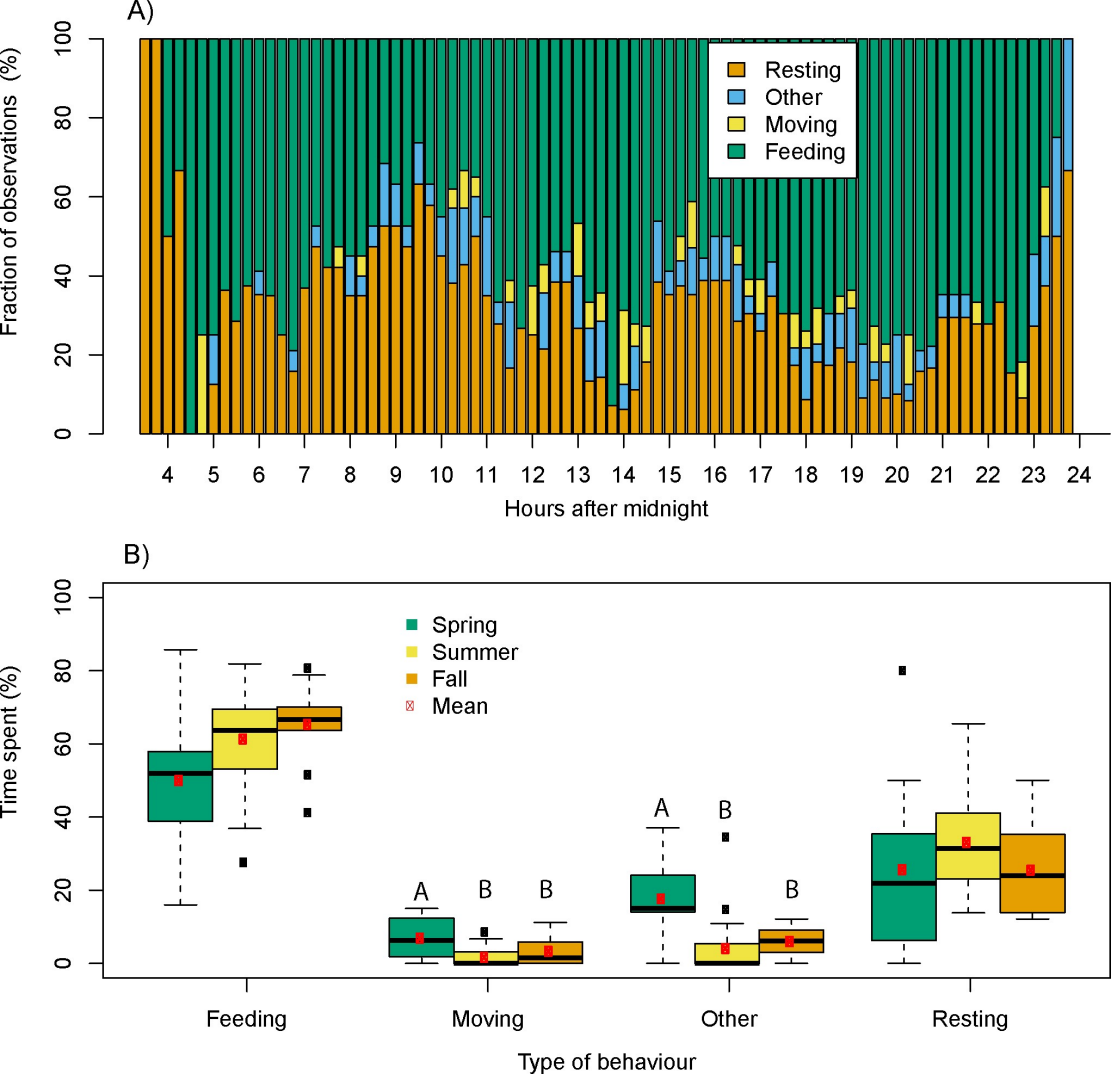
Daily and seasonal behavioural patterns. Fraction of observations where the bison herd rest, feed, move or express other types of behaviour during a day throughout the observation period (spring, summer and autumn) (A). Time spent on a specific type of behaviour across the seasons (spring, summer, and autumn) (B). Different letters are attributed to seasons that vary significantly from other seasons within the same type of behaviour.

**Fig 4:**
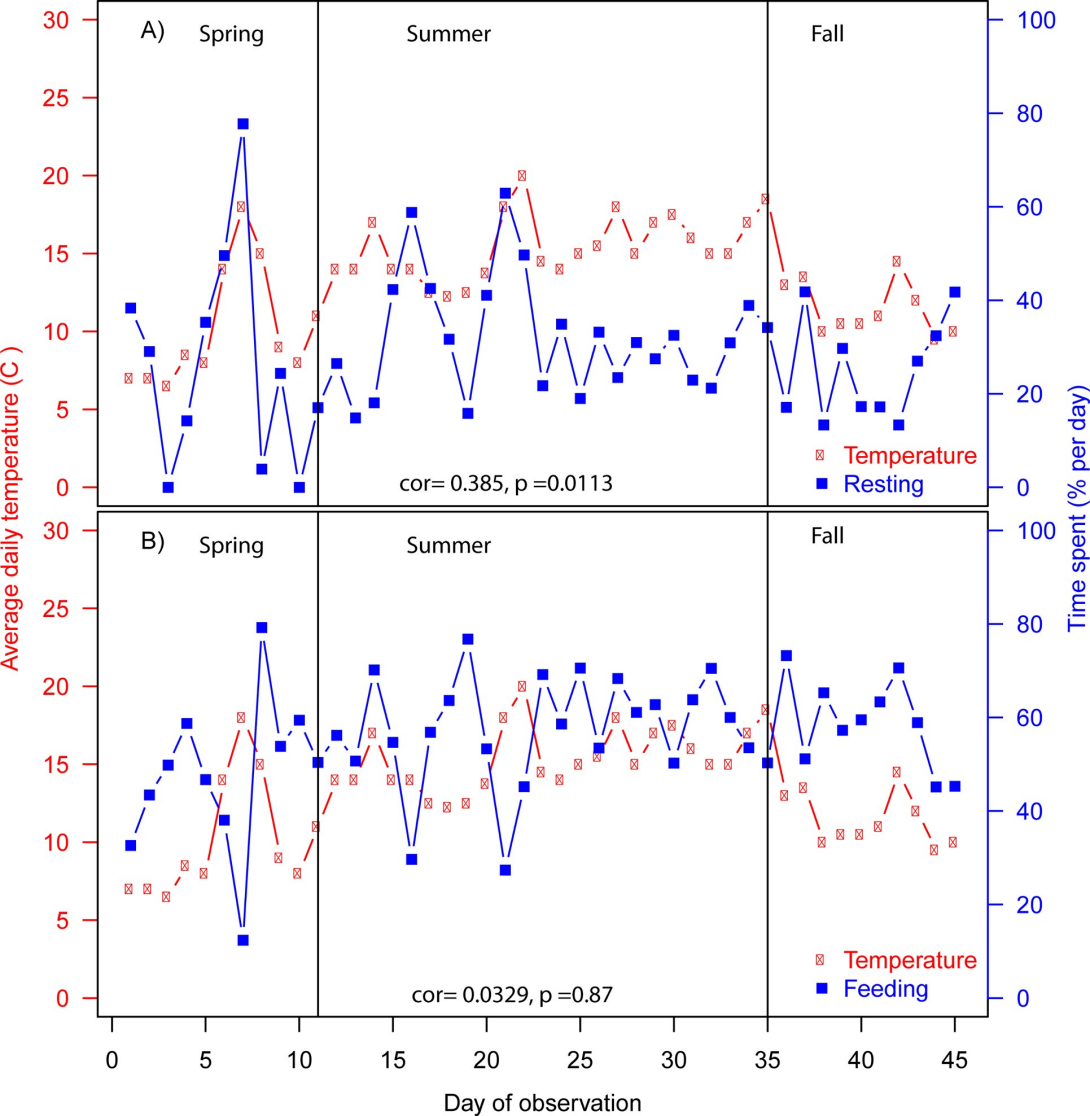
Correlation between temperature and time spent resting or feeding. Correlation between mean daily temperature and time spent on (A) resting or on (B) feeding across the observation period. Statistical results from Pearson´s correlation corrected for temporal correlation are shown.

The diurnal behaviour pattern showed that the bison herd had three major feeding bouts a day (green bars in Fig 3A), namely during 4.30–8.30, 11–15 and 17–23 hours, with feeding bouts defined as periods with 15 minute intervals where more than 50% of the observations over the study period were feeding observations. Generally, the bison herd was resting between the feeding periods (orange bars in Fig 3A). Over the entire growth season, the bison herd spent on average 59.4% on feeding, 29.5% on resting, 3.3% on moving, and 7.8% on other activities (Fig 3B). This activity budget was consistent with previous findings by Caboń-Rackzyńska et al. [33] (χ^2^_2_ = 1.5482, p = 0.461). Time spent on resting did not change significantly over the three seasons (spring, summer, and autumn) (H_2_ = 3.198, p = 0.202, Kruskal Wallis Rank Sum test) (Fig 3B), though time spent on resting was significantly correlated with mean daily temperature (r = 0.38, F_40.58_ = 7.04, p = 0.0113, corrected Pearson correlation for temporal autocorrelation) (Fig 4A). There was a non-significant tendency for the bison herd to spend more time on feeding as the growth season progressed (H_2_ = 5.48, p = 0.0647, Kruskal Wallis Rank Sum test) (Fig 3B). Time spent on feeding and daily temperature were not correlated (r = 0.03, F_23.7758_ = 0.0257, p = 0.87) (Fig 4B). Time spent on moving and other behaviour types showed a significant change across season with the spring period showing significantly higher moving activity than the summer period (p = 0.0020, Dunn´s test) as well as a higher level of other behaviour (e.g. scratching and playing) during spring time than the summer and autumn period (p<0.0001 and p = 0.0217, respectively, Dunn´s test) (Fig 3B).

### 3.2 Habitat use

Overall, the bison herd spent significantly more time in the open meadow than in any of the other types of habitat (cultivated release area, tree covered patches, and open water (χ^2^_3_ = 2101.5, p<0.001) (Table 3), but not as much time as predicted from the availability of the open meadow area (Table 4). Bison spent almost the quadruple amount of time in the release area than expected from its´ availability (Table 4), which was significantly more than time spent in other habitats; meadow, areas with tree cover, and significantly more than in the other management area (meadow with patches of open water and tree covered patches) (Table 3). This selection of the release area is also reflected in the highest Jacob index (0.26) (Table 3). There was no difference in habitat use of meadow habitat and areas with tree cover or between open areas (meadow combined with release area) and areas with tree cover (Table 3).

**Table 3:** Statistical results of chi-square test of habitat use of different habitats and management areas when accounting for their habitat availability.

Habitat use among habitats and management areas	Test results
Habitats: **Cultivated field (Release area)** vs. meadow	χ^2^_1_ = 34.732, p<0.001
Habitats: **Cultivated field (Release area)** vs. tree covered areas	χ^2^_1_ = 14.171, p<0.001
Habitats: meadow vs. tree covered areas	χ^2^_1_ = 0.0020587, p = 0.9638
Habitat: open habitat (cultivated field + meadow) vs closed habitat (tree covered areas)	χ^2^_1_ = 0.20878, p = 0.6477
Management area: **Release area (cultivated field)** vs. meadow with tree and water patches	χ^2^_1_ = 35.492, p<0.001

Bold letters indicate the type of habitat and management area that the bison significantly favors when accounting for habitat availability.

**Table 4:** Time spent by the bison herd in each type of habitat compared to the availability of the habitat types including Jacob´s food selection index.

	Meadow	Tree cover	Cultivated grassland (Release area)	Open water
Habitat used (%), r*100	71.6	9.4	19.0	0
Habitat availability (%), p *100	79.7	10.3	5.2	4.8
Jacob´s index, D	−0.22	−0.05	0.26	−1

**Fig 5:**
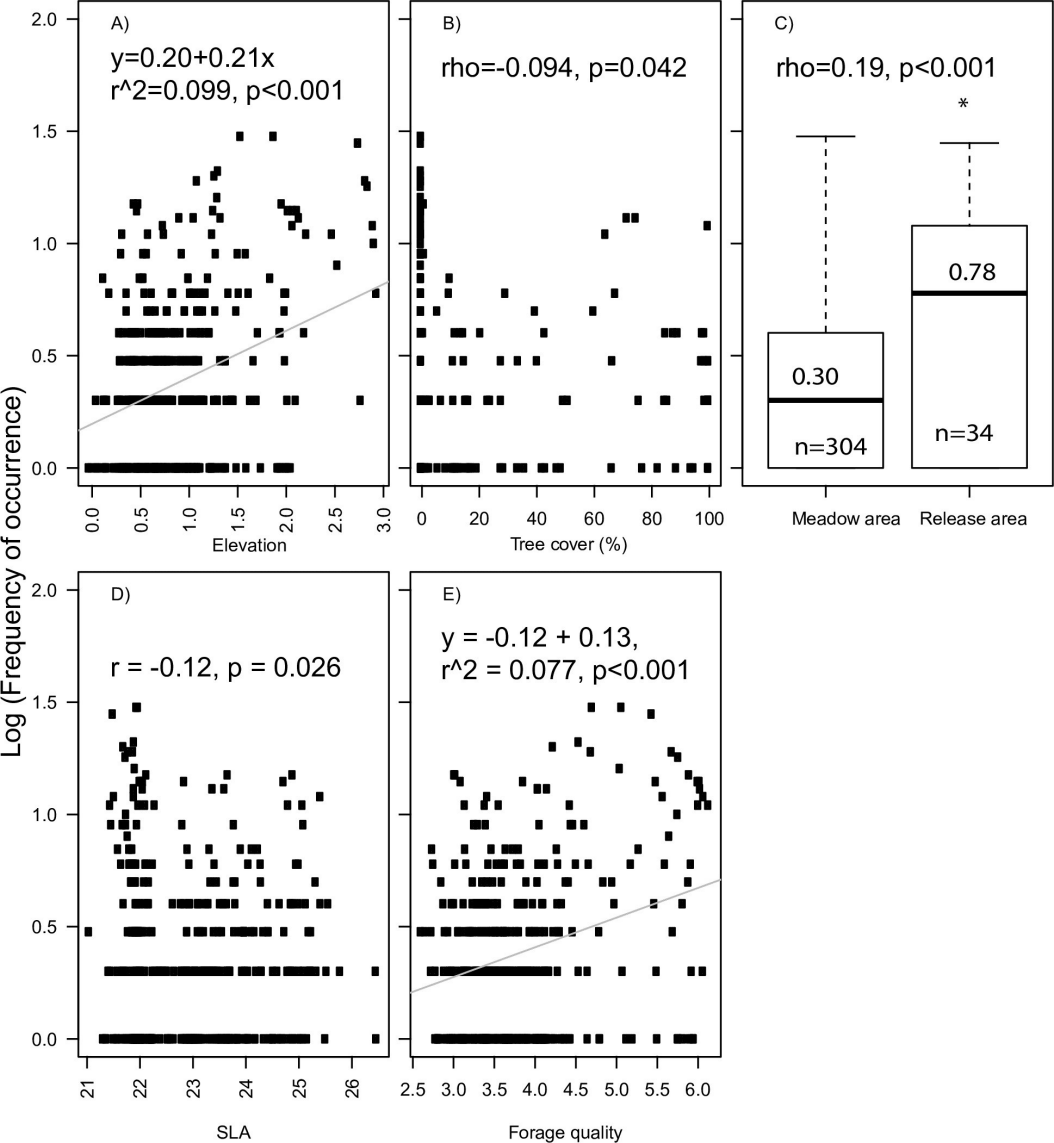
Correlations between frequency of occurrence and predictor variables. Correlations between log frequency of occurrence and (A) elevation, (B) tree cover, (C) management area, (D) SLA, and (E) forage quality). Statistical results from Pearson´s correlation or Spearman´s rank correlation is shown for continuous variables and ordinal variables. An asterisk indicates a significant difference among management areas (C).

**Fig 6:**
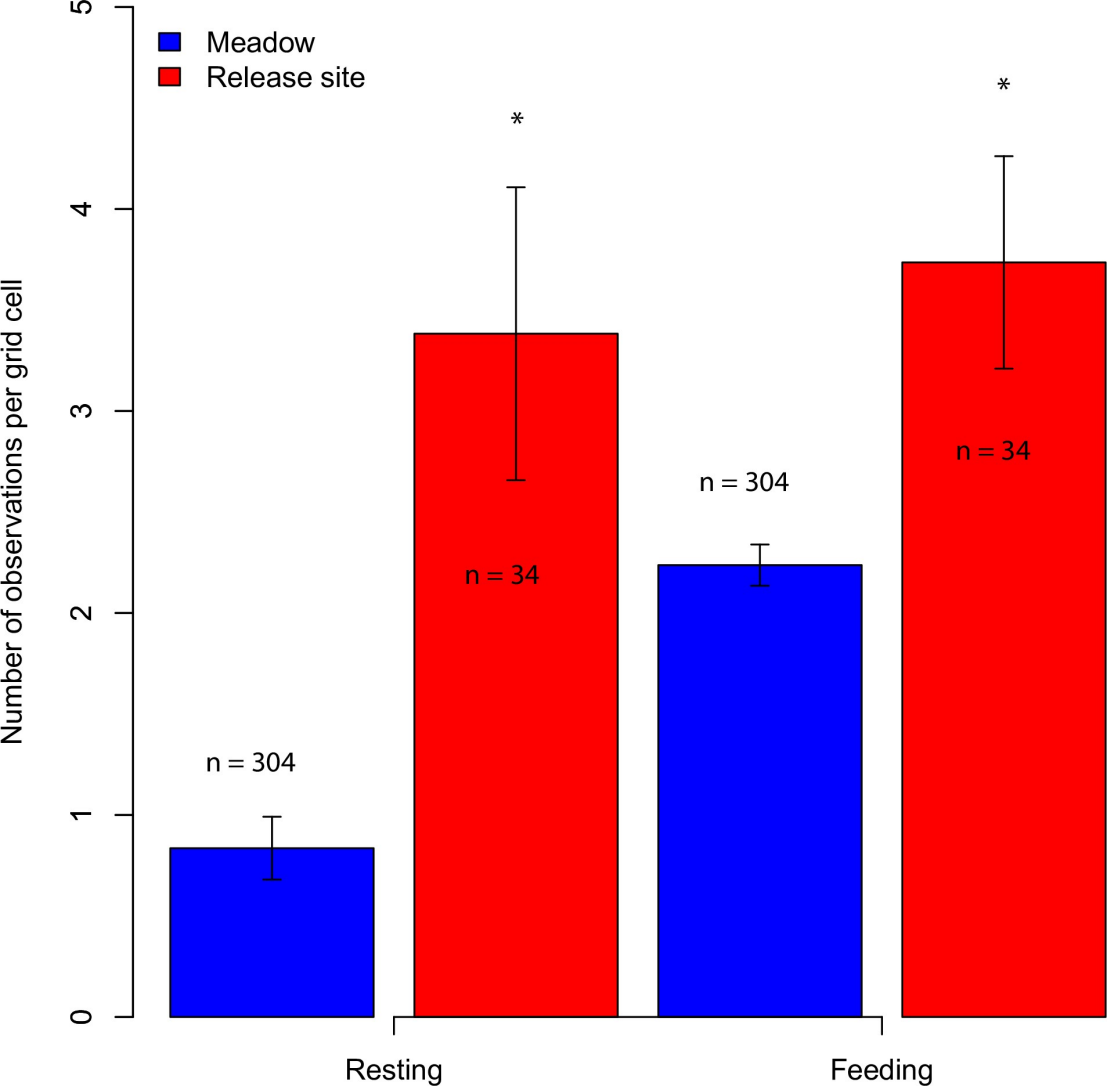
Time spent feeding and resting across management area. Number of feeding events or resting events of the bison herd observed in the meadow area or in the release area. n value indicates number of grid cell per management area. An asterisk indicates a significant difference in number of observations between management area.

Log frequency of occurrence was significantly correlated with management type (Spearman´s rank correlation: rho = 0.19, S = 5211000, p<0.001) (Fig 5) and the number of resting and feeding observations per grid cell in the release area was significantly higher than in the meadow area (Wilcoxon w = 3534, p<0.0001 and Wilcoxon w = 3678.5, p<0.005) (Fig 6). We found no strong relationship between log frequency of occurrence and any explanatory variables, though there were moderate to weak relations to elevation (r^2^ = 0.099, p<0.001), SLA (r = -0.12, p = 0.026) and forage quality (r^2^ = 0.077, p<0.001) (Fig 5).

### 3.3 Multiple linear and logistic models of habitat selection

None of the models considered showed problematic levels of spatial autocorrelation. Therefore, spatial linear models were performed with Ordinary Least Square and spatial logistic models were performed with Generalized Linear Model (S2 Fig). The three best linear regression models (ΔAIC_c_ < 2) all have fairly low w_i_ values and low adjusted r^2^ values (Table 5), indicating that the models neither are notably better than the worst models nor have great explanatory support. These linear regression models indicate that Elevation is the most important predictor as it appears in all the best three models and has the highest effect in the linear regression models, while tree cover and SLA both have minimal effect (Table 6). Elevation had positive coefficients (Table 5), suggesting that bison preferred staying in areas with higher elevation, which were also drier than the area with lower elevation.

**Table 5:** Three best spatial linear models on log frequency of occurrence (ΔAIC_c_ <2) selected from eight tested models and ranked by AIC_c_.

Model coefficients (n = 1095)			ΔAIC_c_	w_i_	Adjusted r^2^
Tree cover	Elevation	SLA			
−0.08	0.37		0	0.317	0.141
	0.37		0.27	0.277	0.138
−0.10	0.42	0.08	1.73	0.273	0.143

AIC_c_ (corrected Akaike Independent Criterion); w_i,_ Akaike weights.

**Table 6:** Summary of results after full model-averaging: Effects of each explanatory variable on frequency of occurrence (linear regression) and presence/absence (logistic regression) of bison.

Predictors	Linear regression				Logistic regression		
	Estimate	Adj. SE	CI 95%	RVI	Estimate	Adj. SE	CI 95%
Elevation	0.391	0.0587	0.275 – 0.506	1	0.435	0.227	−0.083 - 0.807
SLA	0.0291	0.0545	−0.0777 - 0.136	0.59	-1.28	0.221	−1.71 - −0.844
Tree cover	−0.0514	0.0592	−0.167 - 0.0647	0.41	0.630	0.159	0.318 - 0.941

Adj. SE, Adjusted standard error; CI 95%, 95% confidence interval; RVI,relative variable importance.

For the logistic regression models, the best models seemed to have much higher explanatory support than the second best model (w_i_ = 0.85 vs. w_i_ = 0.15, respectively), though both had low predictive power (McFadden’s r^2^ = 0.0830 and 0.0790). All explanatory variables were included in the best model, whereas the second best model did not include elevation. Elevation and tree cover had positive coefficients, whereas SLA had negative coefficients (Table 7). SLA had the highest effect for the logistic regression models, and tree cover and elevation had moderate effects (Table 6). The logistic model indicates that the bison herd more likely occurred in areas with higher elevation, like the linear regression model, but also in areas with lower SLA-values and more tree cover. Overall, these models suggest that the bison herd visited high-lying, drier areas more often than low-lying areas, wet areas, and that the bison herd less likely visited areas with high SLA-values at all.

**Table 7:** The best spatial logistic model on presence/ absence occurrence (ΔAIC_c_ <2) selected from eight tested models and ranked by AIC_c_.

Model coefficients (sample size: n = 338)			ΔAIC_c_	w_i_	McFaddens r^2^
Tree cover	Elevation	SLA			
0.61	0.42	−1.2	0	0.85	0.0830
0.72		−1.5	3.47	0.15	0.0790

AIC_c_, (corrected Akaike Independent Criterion); w_i_, Akaike weights.

**Fig 7:**
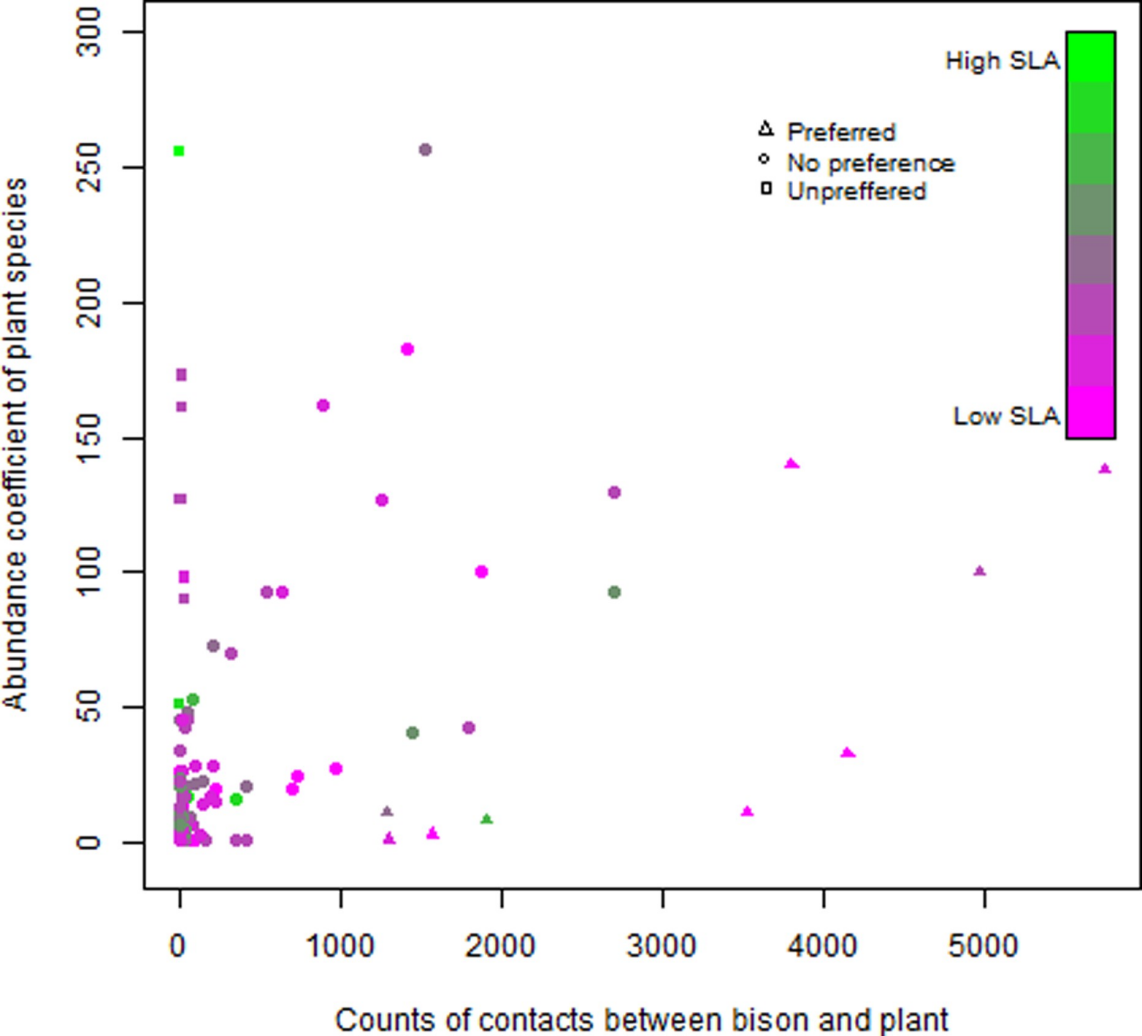
Relation between plant preference and SLA-values. Correlation between plant-bison contacts, availability of plant species, and SLA-values of plant species. **Un-preferred plant species** (rectangle) is characterized by being abundant while having few contacts with a bison, **Preferred plant species** (triangle) is characterized by having a low abundance while having many contacts with a bison, and **plant species with no preference** (circle) is characterized by having increasing contacts with a bison with increasing abundance. Figure adapted from Borowski and Kossak, 1972.

Assigning SLA values to the bison food preference data obtained by Borowski and Kossak [49] allowed us to test the relationship between food preference and SLA (n/D~SLA) (Fig 7). The linear regression had very low explanatory power and SLA was a poor predictor of food preference (adjusted r^2^ = 0.00082, p = 0.30).

## 4. Discussion

### 4.1 Behavioural patterns

In this study, we wished to assess the daily behaviour of bison and compare with previous findings in Bialowieza Forest. The bison herd had three major feeding bouts a day (Fig 3A). In comparison, previous studies from Bialowieza Forest report four feeding bouts a day in periods without snow cover [33] and in periods with snow cover two feeding bouts have been reported [35]. On average the bison herd spent 59.4% on feeding, 29.5% on resting, and 3.3% on moving across the growth season (Fig 3B), which was consistent with previous findings on bison in Bialowieza Forest [33]. This is an interesting finding as time spent on feeding is thought to depend on forage quality and quantity [51] indicating that the food quality and quantity in this enclosure is equally good as the food available for the free-roaming bison in Bialowieza. Bison reintroduced in the Alps in France with access to supplementary fodder spent more than 40% of the time feeding and approximately 50% resting independent of snow cover [18]. Caboń-Rackzyńska [35] found that bison in Bialowieza forest, also with access to supplementary fodder, spent even less time of the day feeding (30%) and correspondingly spent more time resting in periods with snow cover. Both these studies support that fodder with presumably high food quality reduce the overall time needed to feed. Resting more during cold periods can also be related to shifts in temperature and weather conditions, which appear to cause the European bison to behave more docily [33]. Our observations also support that shifts to higher temperature increase the amount of time spent on resting (Fig 4A).

The bison herd in Vorup meadows overall seem to behave like free-roaming bison observed in large forested areas less human-modified, despite being confined to a heavily human-modified meadow enclosure. This is an important finding as this indicates that rewilding with European bison even in anthropogenic environments can support thriving animals expressing what we, so far, believe is natural behaviour in the wild. Knowing this also enables us to transfer knowledge from one reintroduction site to another more confidently.

### 4.2 SLA is a poor predictor for habitat selection

In the logistic regression models, SLA had a higher effect than elevation (Table 7), suggesting that SLA was a better predictor of presence/ absence occurrence than elevation and that SLA values often were low where the bison herd occurred. Whether bison actually avoided areas with high SLA values or not, is hard to deduct as elevation and SLA were 59% correlated (See S1 Table), just below the threshold for exclusion based on multicollinearity, and therefore the signals are hard to distinguish. Several of the herbaceous plants that European bison is reported to prefer (e.g. *Urtica diodica*, *Cirsium oleracium*, *Plantago sp*, *Filipendula ulmaria*) [52] were available in the enclosure, and in several vegetation plots, we observed clear bite marks on these species. Plants with high SLA values observed in the enclosure included e.g. *Impatiens capensis* (SLA 54.3 mm^2^/mg), *Urtica dioca* (SLA 31.6 mm^2^/mg), *Alisma plantago-aquatica* (SLA 29.7 mm^2^/mg), *Cirsium oleracum* (SLA 25.7 mm^2^/mg), and several grasses (SLA values from 18–35 mm^2^/mg). Some of the tree species (e.g. *Betula pubescens*, *Alnus glutinosa*, *Salix* spp.) reported as preferred [52] were also observed in the enclosure and showed clear signs of debarking. These species had lower SLA values than many herbaceous plants. One might think that plant species with high SLA values might be attractive for foraging bison due to their seemingly high palatability, but in this study, the logistic models indicated a negative relationship between occurrence and SLA. Testing the relationship between SLA and food preference for plant species (n/D) obtained by Borowski and Kossak [49] studying bison in Bialowieza Forest showed that SLA had very low explanatory power for food preference (adjusted r^2^ = 0.00082, p = 0.30) (Fig 7). These results suggest that SLA is a poor predictor for the habitat selection of bison.

### 4.3 Habitat use

We found that bison used the release area more than expected from availability, but did not favour open habitat over forested habitat when accounting for habitat availability. It is not clear whether the preference for the release area is linked to its inherent ethological value (i.e. the possible status of the release area as their “home” might influence their behaviour accordingly), or to the rather high forage quality of the release area (Fig 2C) due to former grass sowing management or to the availability of supplementary fodder, or that the release site was placed at the driest part of the enclosure. It is reasonable to think that the preference for the release area is linked to feeding habits as the herd was observed significantly more times feeding in grid cells in the release area than in the meadow (Fig 6), however, the herd was also observed significantly more times resting in grid cells in the release area than in the meadow indicating that the release area has ethological value for the bison (Fig 6). During the study period, bison were observed feeding close to the hay rack more often than in the rest of the release area (mean number of feeding observation in grid cells with hay rack vs the rest of the release area: 4.2 vs. 4>), however, bison were only observed feeding directly from the hay rack a few times in the total study period (silage was only provided occasionally during the study period). This indicates that bison exploited the supplementary fodder minimally, but more likely were attracted to the release area due to the high forage quality, (Fig 5), dry habitat (Fig 5) or the release event. In the following section we discuss the potential role of the release event, fodder supply and elevation (moisture) on habitat use.

Schmitz and colleagues recently investigated the exploration behaviour of a European bison herd reintroduced to a mountainous area in Germany during the transition from an 89 ha release area (occupied for three years) to the total designated area of 4500 ha. They found that the release area was fully or partially included in the 85% Kernel density (i.e. used areas) by the herd or the bull in 15 out of 18 10-days periods during the first 6 months of exploration [19]. Though the authors explicitly state that bison only used the release area occasionally, these results indicate to us that the exploration behaviour by bison is centred around the release area in the majority of the time during the first 6 months and that the release area, like in our study, plays a significant role in how new habitats are occupied.

Evidence exists from other studies that European bison show higher habitat preference for areas provided with supplementary fodder than if no supplementary feeding takes place [53, 54]. Recently Ramos and colleagues [18] showed that a herd of European bison reintroduced in the French Alps spent more than 50% of the time in areas with supplementary fodder, but when no provision of fodder occur bison spent significantly less time by the food racks and exploited other areas to a higher degree [18]. Influence of access to supplementary fodder on bison winter diet was recently investigated using DNA-barcoding by Kowalczyk and colleagues in Bialowieza Forest. Kowalczyk et al. [55] reported for bison occurring in forest habitats that woody material made up 16% of the diet of bison intensively fed with supplementary fodder, whereas the diet of non-fed bison was composed of 65% woody material [55].

Kerley and colleagues [23] have found that more than two-thirds of the free-ranging bison herds have expanded their range, from originally being forest habitat, to also include open habitats. This, however, is not considered a result of the bison´s habitat preference, but as a result of a management problem as the open habitats bison adopt often are agricultural lands. Conspicuously, 65% of the free-ranging bison herd are also provided with supplementary fodder in order to reduce unintended impacts of bison herds [23]. Kerley and colleagues [23] have suggested that the overall larger habitat use of forest by bison observed today is a consequence of anthropogenic pressure, habitat change, and the fact that conservation efforts have worked within the “bison as a forest species” paradigm.

Elevation is for the study area related to wetness of the soil, as the enclosure is placed on the Gudenå River bank, with terrain rising up away from the water. The area is in the lower parts susceptible to flooding, which could be the reason why the bison herd seems to prefer higher grounds. In this study we did not observe the bison herd occupying the open water patches one single time; however, during the floristics analysis we saw obvious bite marks in areas partly submerged and bison have also been observed in water-filled ditches. van Vuure has pointed out that bison´s preference for drier areas is reflected in the distribution of fossils, where only a few findings occur in river sediments [56]. However, there are studies indicating that bison prefer moist habitats like the study by Daleszczyk and colleagues [57] where bison was found to prefer the moist deciduous forest above all other less moist types of forests in the Polish Bialowieza forests or the recently published study by Zielke et al. [58] documenting that bison spent more time in wet open habitats in the German rewilding area Doeberitzer Heide than expected from availability. Previous studies looking into habitat use or selection and food selection of bison have not incorporated terrain in their analyses (e.g. [18, 19]). In a global study, Olff et al. [59] linked plant-available nutrients and soil moisture to large herbivore densities and suggested based on this that large herbivores prefer drier areas as forage quality of plants are higher due to higher availability of soil nutrients. Unfortunately, in this study, we could not investigate the spatial relationship between habitat use and forage quality as forage quality was excluded from the spatial analysis due to multicollinearity between the elevation and the forage quality.

When bison graze and spend time at the food rack or grazing the former cultivated field they cannot exert their ecological function in other areas at the same time. This means that the ecological impact by bison is reduced not only in terms of grazing pressure but also in terms e.g. browsing, debarking [17, 55], trampling, distributing autochtonous nutrients, and seed dispersal, presumably lowering the potential of the bison to affect plant diversity, structure and composition, and thereby species communities dependent on these e.g. arthropods, amphibians, reptiles, birds and mammals [10]. This might compromise or oppose conservation goals of trophic rewilding initiatives focusing on ecological restoration based on restoring trophic top-down interactions and associated cascades as well as non-feeding related processes of the European bison [22]. Therefore, in terms of rewilding category according to TRAAIL [34] (categorised to be partial rewilding based on ambitions) the results from this study questions whether the bison herd can advance the area into a self-regulating ecosystem as the bison seem to be attracted to the release area due to the high forage quality here or the release event, which turns the project into a minimal rewilding project.

Reintroductions are often conducted in two steps; releasing the bison into to a smaller release area e.g. with supplementary fodder initially or continually, followed by allowing animals access to an adjoining, larger, and often more natural enclosure area [19]. Based on the results presented in this study and other recent studies of bison reintroductions [18, 19, 55] we advocate that constellation of release area and final reintroduction area should be thoroughly considered along with how management protocols (e.g. supplementary fodder) might affect the habitat use by the animals and thereby conservation goals–particularly when these are framed within a trophic rewilding context.

## 5. Conclusion

Overall the present study found similar daily and seasonal behavioural patterns and habitat selection of a semi-free-ranging bison herd as seen in studies on free-living herds, despite the small living space and confinement and a high degree of human modification. These results indicate that rewilding with European bison in anthropogenic environments can support what appears to be natural behaviour. This is highly relevant as bison increasingly are being reintroduced to confined enclosures in anthropogenic landscape in order to 1) help conserve the species and 2) restore ecological functions of the ecosystem, which might be compromised if natural behaviours are not expressed.

The unexpectedly high use of the release site compared to the rest of the enclosure prompt us to raise awareness of the possible long-term ethological effects of the release site and the management protocols accomplished here, as this might cause the ecological impact by bison to be reduced in terms of feeding and non-feeding activities. Further, this might reduce the potential of the bison to affect plant diversity, structure and composition, and thereby species communities dependent on these, and thereby compromise or oppose the conservation goals addressed in a trophic rewilding context. We, therefore, encourage managers to carefully consider the management in the release area relative to the overall aims of reintroducing bison in the particular reintroduction area.

European bison is classified as vulnerable by IUCN and therefore still more populations are needed. Combining large-scale rewilding and bison reintroduction might well be a win-win situation, as large-scale rewilding can support bison population and help ensure genetic diversity, and bison can contribute functionally to rewilding projects especially when framed within a trophic-rewilding context. In line with Schneider [60] we think that in order to further nature conservation, as well as the conservation of European bison, more research is needed to assess to what degree European bison can replace conventional management protocols such as mowing and to determine adequate habitat settings for the European bison.

## Supporting information

S1 Table Test of multicollinearity among predictor variables. Multicollinearity of predictor variables considered for use in spatial linear and logistic models. A correlation (r value) of 0.60 was considered a threshold for including variables in the spatial model analyses.
(DOCX)

S2 Table Daily rhythm. Percentage of observations designated to feeding, resting, moving and other behaviour during a day across the observation period.
(CSV)

S3 Table Seasonal behaviour. Percentage of observations designated to feeding, resting, moving and other behaviour across seasons.
(CSV)

S4 Table Behaviour and temperature. For each observation day, date, time of sun rise and sun set, daily average temperature and time spent on feeding, resting, moving and other behaviours is given.
(CSV)

S5 Table Behaviour across management areas. For each grid cell in the bison enclosure management area, type of behaviour and frequency of occurrence is given.
(CSV)

S6 Table Habtat use. For each grid cell coordinates, values of environmental predictor variables, number of bison occurrences in total and divided into behaviour types is given.
(CSV)

S1 Fig Seasonal temperatures. Daily average temperature of spring, summer, and autumn. Different letters indicate statistical significant differences in daily average temperature among season.
(PDF)

S2 Fig Spatial autocorrelation. Evaluation of spatial autocorrelation based on Moran´s I of first 20 distance classes of model residuals of simultaneous autoregressive lagged (SAR lag) models, simultaneous autoregressive error (SAR err) models, Ordinary Least Square (OLS) regression and Generalized Linear Models (GLM).
(PDF)
