# Supplementary material for: Wild Steps in a semi-wild setting? Habitat selection and behavior of European bison reintroduced to an enclosure in an anthropogenic landscape
Source: PLoS One. 2019 Nov 7;14(11):e0198308. doi: 10.1371/journal.pone.0198308 (PMC6837835; doi:10.1371/journal.pone.0198308)
Supplement: S1 Table — Multicollinearity of predictor variables considered for use in spatial linear and logistic models. A correlation (r value) of 0.60 was considered a threshold for including variables in the spatial model analyses. (DOCX) [file pone.0198308.s001.docx]

# S1 Table

Multicollinearity of predictor variables considered for use in spatial linear and logistic models. Correlation (r value) above 0.60 were exclude from spatial model analysis.

|  | DTM | Tree cover | Management  area | SLA | Forage Quality |
| --- | --- | --- | --- | --- | --- |
| DTM | 1 |  |  |  |  |
| Tree cover | 0.25 | 1 |  |  |  |
| Management  area | 0.72 | -0.14 | 1 |  |  |
| SLA | -0.59 | 0.093 | -0.38 | 1 |  |
| Forage Quality | 0.89 | 0.16 | 0.77 | -0.73 | 1 |
