# Supplementary figures and images for: Wild Steps in a semi-wild setting? Habitat selection and behavior of European bison reintroduced to an enclosure in an anthropogenic landscape
Source: PLoS One. 2019 Nov 7;14(11):e0198308. doi: 10.1371/journal.pone.0198308 (PMC6837835; doi:10.1371/journal.pone.0198308)

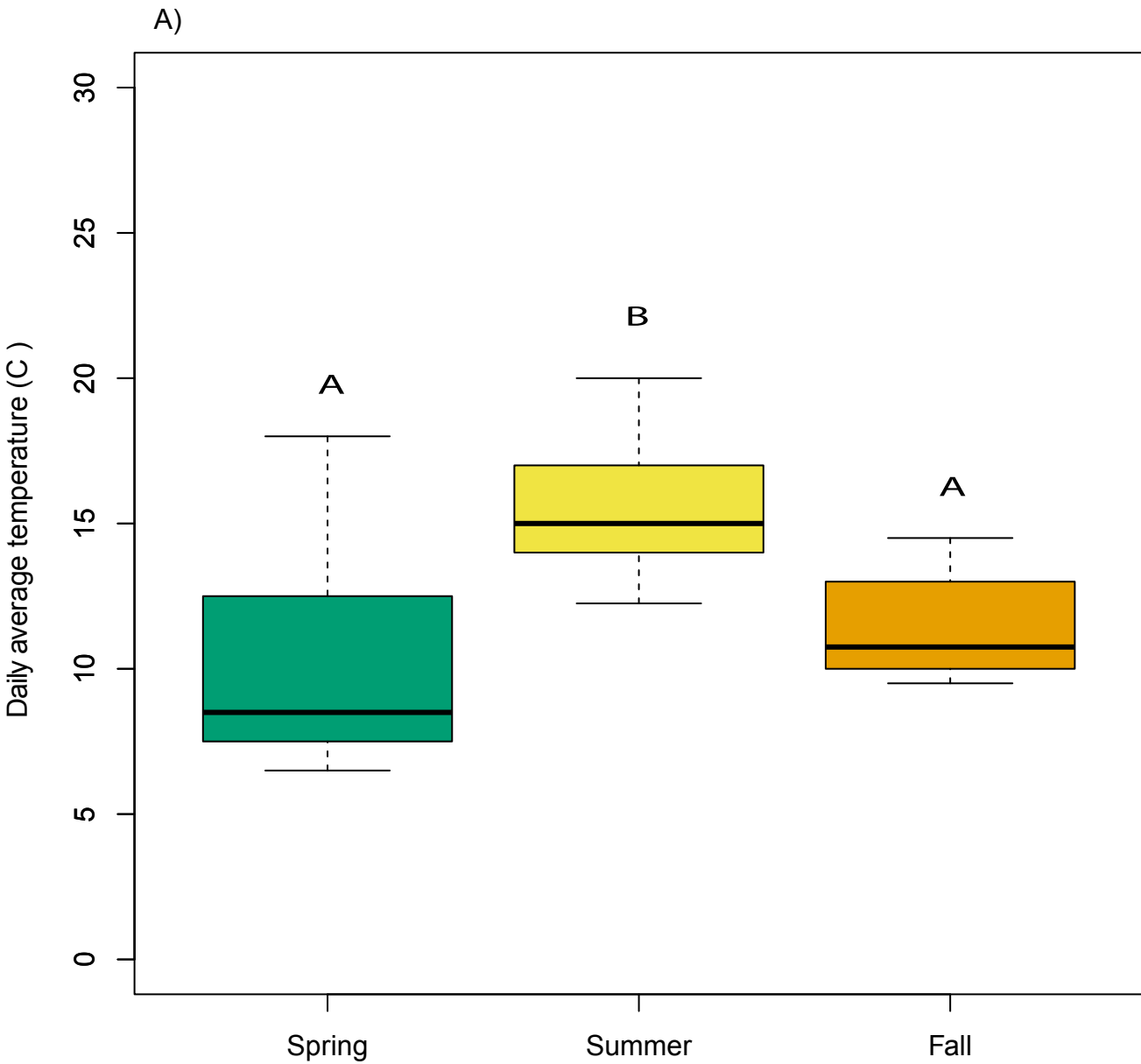

Supplement: S1 Fig — Daily average temperature of spring, summer, and autumn. Different letters indicate statistical significant differences in daily average temperature among season. (PDF) [file pone.0198308.s007.pdf]

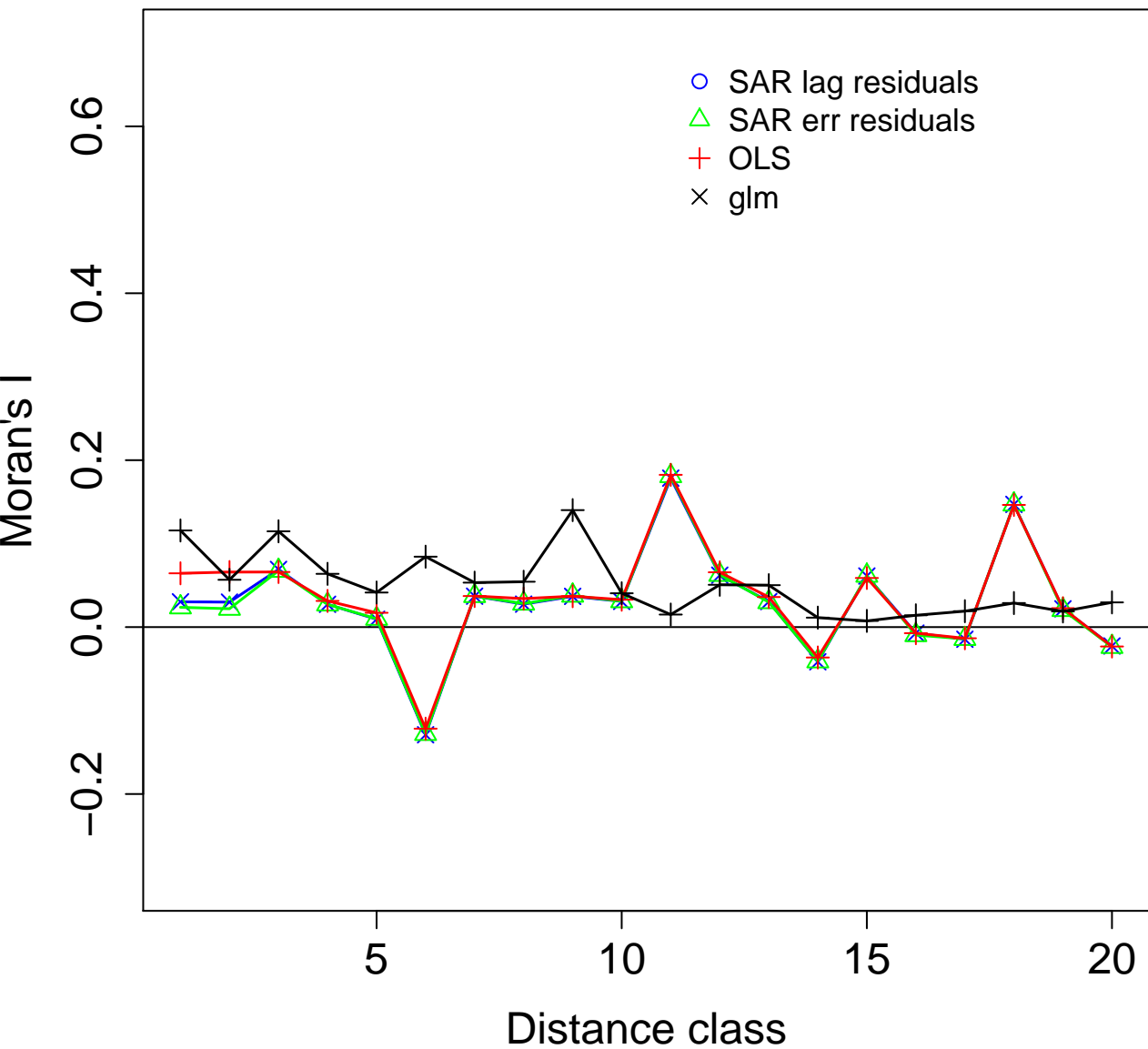

Supplement: S2 Fig — Evaluation of spatial autocorrelation based on Moran´s I of first 20 distance classes of model residuals of simultaneous autoregressive lagged (SAR lag) models, simultaneous autoregressive error (SAR err) models, Ordinary Least Square (OLS) regression and Generalized Linear Models (GLM). (PDF) [file pone.0198308.s008.pdf]
